# Objective evidence for chronic back pain relief by Medical Yoga therapy

**DOI:** 10.3389/fpain.2022.1060685

**Published:** 2022-12-23

**Authors:** Suvercha Arya, Raj Kumar Yadav, Srikumar Venkataraman, Kishore Kumar Deepak, Renu Bhatia

**Affiliations:** ^1^ Pain Research and Transcranial Magnetic Stimulation, Laboratory, Department of Physiology, All India Institute of Medical Sciences, New Delhi, India; ^2^ Integral Health Clinic, Department of Physiology, All India Institute of Medical Sciences, New Delhi, India; ^3^ Department of Physical Medicine and Rehabilitation, All India Institute of Medical Sciences, New Delhi, India; ^4^ Autonomic and Vascular Function Testing Laboratory, Department of Physiology, All India Institute of Medical Sciences, New Delhi, India

**Keywords:** chronic low back pain, Medical Yoga therapy, nociceptive flection reflex, central sensitisation pain, diffuse noxious inhibitory control (DNIC), conditioned pain modulation (CPM)

## Abstract

Chronic low back pain (CLBP) is a musculoskeletal ailment that affects millions globally. The pain is disturbing associated with impaired motor activity, reduced flexibility, decreased productivity and strained interpersonal relationships leading to poor quality of life. Inflammatory mediators in vicinity of nociceptors and amplification of neural signals cause peripheral and central sensitization presented as hyperalgesia and/or allodynia. It could be attributed to either diminished descending pain inhibition or exaggerated ascending pain facilitation. Objective measurement of pain is crucial for diagnosis and management. Nociceptive flexion reflex is a reliable and objective tool for measurement of a subject's pain experience. Medical Yoga Therapy (MYT) has proven to relieve chronic pain, but objective evidence-based assessment of its effects is still lacking. We objectively assessed effect of MYT on pain and quality of life in CLBP patients. We recorded VAS (Visual analogue scale), McGill Pain questionnaire and WHOQOL BREF questionnaire scores, NFR response and Diffuse noxious inhibitory control tests. Medical yoga therapy consisted of an 8-week program (4 weeks supervised and 4 weeks at home practice). CLBP patients (42.5 ± 12.6 years) were randomly allocated to MYT (*n* = 58) and SCT groups (*n* = 50), and comparisons between the groups and within the groups were done at baseline and at end of 4 and 8 weeks of both interventions. (VAS) scores for patients in both the groups were comparable at baseline, subjective pain rating decreased significantly more after MYT compared to SCT (*p *= < 0.0001*, *p* = 0.005*). McGill Pain questionnaire scores revealed significant reduction in pain experience in MYT group compared to SCT. Nociceptive Flexion Reflex threshold increased significantly in MYT group at end of 4 weeks and 8 weeks, *p* < 0.0001#, *p *= < 0.0001∞ respectively) whereas for SCT we did not find any significant change in NFR thresholds. DNIC assessed by CPT also showed significant improvement in descending pain modulation after MYT compared to SCT both at end of 4 and 8 weeks. Quality of life also improved significantly more after MYT. Thus, we conclude with objective evidence that Medical Yoga Therapy relieves chronic low back pain, stress and improves quality of life better than standard care.

## Introduction

Chronic Low Back Pain (CLBP) is defined by National Institute of Health (NIH) Pain Consortium, as a back pain problem that has persisted for at least 3 months and has resulted in pain on at least half the days in the past 6 months (1). The cardinal features of CLBP are chronicity, perturbed sensory information transfer and processing, impaired motor activity and incongruence of somatosensory interactions leading to unfavorable cortical plasticity. Low back pain is a significant public health problem, with huge socioeconomic costs, predisposing patients at significantly higher risk of depression, anxiety and strained interpersonal relationships. The condition characterized by a hyper arousal state of nervous system, increased tension of the affected musculature, altered patterns of breathing, low energy levels and overall a poor mindset. The physical and emotional stressors exacerbate the distress and overall quality of life of the patient (2). CLBP is typically associated with chronic inflammation, and undue activation of somatosensory, immune, neuronal, autonomic, and circulatory systems (3). Nociceptors are sensitized by inflammatory mediators present in the vicinity such as prostaglandins, nerve growth factor, bradykinin and pro-inflammatory cytokines leading to peripheral sensitization. Prolonged duration of pain also causes maladaptive neuroplasticity (4–6) and altered neurotransmitter concentrations (7–9) at spinal and supraspinal levels of the nociceptive system. Chronicity eventually leads to central sensitization of dorsal horn neurons manifested as prolonged neuronal discharges, increased responses to defined noxious stimuli (hyperalgesia), response to non-noxious stimuli (allodynia), and expansion of receptive fields. A dysfunction of the descending inhibitory control/pain modulatory systems also contributes to central sensitization.

Recently, a large number of studies have emerged addressing the management of nonspecific chronic low back pain in form of oral medications [NSAIDs/Coxibs; Opioids; Antidepressant; muscle relaxants; Anti-convulsion medication (for radicular pain), Capsaicin] diathermy, acupuncture, Acupressure, physiotherapy and exercises. However, the recommendations are non-specific and lack disease oriented guidelines that can best alleviate low back pain (10). The American College of Physicians (**ACP**) and American Pain Society (**APS**) in 2007 proposed a joint clinical practice guideline on diagnosing and treating nonspecific low back pain. It recommends adjunct nonpharmacologic therapies to be considered for patients whose conditions do not improve with education, self-care options, or pharmacologic interventions even after 3 months of treatment (11). Another promising mode of management which focuses on physical flexibility and mental relaxation through well-defined postures and manoeuvres is yoga based medical therapy.

Since pain and lack of flexibility are the two important hallmarks of CLBP, ancient techniques such as yoga based intervention have emerged as a unanimous therapy of choice by many scientific groups. Supporting the same, many studies that have reported beneficial effects of Yoga in chronic low back pain but most of them rely on subjective measures to assess the effectiveness of yogic intervention. The commonly used tools include numerical pain rating scores (Visual analogue scale), and various questionnaires like (WHO Quality of Life, McGill Pain, Oswestry's disability index, Back pain bothersome, Back Pain Self-Efficacy Scale, Roland-Morris Disability Questionnaire, Short Form-36 health status, Treatment satisfaction, Beck Depression Inventory, fear avoidance beliefs questionnaire, Perceived Stress Scale, Pain attitudes (SOPA-Survey of Pain Attitudes), Coping (CSQ-R- coping strategies questionnaire-revised), Self-efficacy (BPSES- brief parental self-efficacy scale) (12–22). All these assessment tools are dependent on patient's response that is quite subjective in nature and prone to significant bias.

Therefore, the present study was designed to study the effect of medical yoga therapy on chronic low back pain using objective pain assessment tools, i.e., Nociceptive flexion reflex and Diffuse noxious inhibitory control test.

Nociceptive flexion reflex (NFR), also called as RIII reflex, is an objective, reproducible and elicitable neurophysiological tool to evaluate nociception (23). Physiologically it is a polysynaptic reflex allowing for painful stimuli (noxious) to activate an appropriate withdrawal (flexion) response. The reflex is spinally organized, influenced by an endogenous pain modulating system. It is debatable whether this chronic pain state is due to “bottom up” amplification or “top down” insufficiency mechanisms. The “bottom up” theory suggests increase in pain perception is due to excess noxious peripheral inputs that sensitizes the nervous system to the point of perceiving pain even in absence of peripheral stimulus (24). The “top down” theory suggests changes within the pain modulatory centers are unable to modulate the pain, regardless of peripheral noxious input (25). Central sensitization refers to “an amplification of neural signaling within the central nervous system that elicits pain hypersensitivity” presented as allodynia, and/or hyperalgesia in widespread locations in addition to areas associated with the underlying pain disorder (26). The instigating factor of central sensitization could originate in the periphery through mechanisms that eventually lead to Long-Term Potentiation (LTP) in the spinal cord as well as structural changes in the brain.

Descending noxious inhibitory control test is a tool to assess endogenous pain control system that originates in the brainstem which descends to the spinal dorsal horn where pain modulation takes place. At the dorsal horn, inhibition of nociceptive transmission occurs by release of serotonin and noradrenalin which results in reduction of nociceptive input to the brain (27, 28). Higher cortical areas like the prefrontal/anterior cingulate cortex anatomically and physiologically target the origin of the descending pain inhibitory pathways (29). These areas are involved in cognitive and emotional processing, that makes descending pain inhibition susceptible to cognitive-emotional modulation (30–32).

Compared to healthy adults, patients of chronic pain have repeatedly been shown to exhibit impaired descending pain modulation/pain inhibition, which results in chronicity of pain (33). Thus, improving descending pain modulation in subjects with chronic pain is promising for pain therapy (34).

Therefore the present research was intended to investigate the effect of Medical Yoga Therapy on objective measures of pain assessment in chronic low back pain.

## Material and methods

The research was conducted in the Pain research and TMS Laboratory, Department of Physiology, All India Institute of Medical Sciences (AIIMS), New Delhi. A randomized control trial was designed to compare the effect of Medical Yoga Therapy (MYT) and Standard Care Therapy (SCT) in CLBP patients, the recordings were done at baseline, at end of 4 and 8 weeks of both interventions. The study was approved by Institute Ethics Committee of the AIIMS, New Delhi (Ref.No. IECPG-186/27/27.03.2019, RT-10/27.04.2019) and all procedures were registered on Clinical Trial Registry India (REF/2017/10/015616). Chronic low back pain patients visiting the outpatient department of Physical Medicine and Rehabilitation, Orthopaedics and Neurosurgery departments, AIIMS, New Delhi were screened by specialists. Clinical conditions like malignancy, fractures, ankylosing spondylitis, infections, caudal equina syndrome, radiculopathy were ruled out. Neuropathic pain was also ruled out by straight leg raising test. Chronic low back pain was defined as per the criteria described by the NIH pain consortium non-specific pathology (1). CLBP patients with no other chronic pain condition aged 18 to 65 years were included after thorough screening. The exclusion criteria were; presence of any major illness (psychiatric, neurological, autoimmune, cardiovascular), history of opioid or substance abuse.

Objective and subjective assessment of pain were done using the following measure, VAS scoring, WHOQOL BREF questionnaire, McGill Pain questionnaire, Nociceptive Flexion Reflex (NFR) recording and Diffuse Noxious Inhibitory Control (DNIC) test.

Nociceptive Flexion Reflex recording, site of stimulation was Sural nerve (pure sensory nerve that runs along the retromalleolus of the ankle region). Noxious electrical stimuli were delivered using Bipolar Ag/AgCl electrodes at the skin superficial to the sural nerve, ranging between 10–100Volts. The response to the electrical stimulation was a withdrawal picked up as an electromyographic signal by surface electromyography electrodes placed on the short head of the biceps femoris. The site was identified by palpating the tendon against resistance about 4–5 fingers away from the popliteal fossa. A train of 5–10 square wave electrical pulses at a frequency of 200 Hz with 1 msec duration were delivered. Each consecutive stimulus was separated by 5–15 s. The test started with a familiarity session by giving the participant a wide range of electrical stimuli. A step-up method in jumps of 5Volts was employed during recording of the NFR threshold. Thereafter, the intensity was decreased by 1Volt to confirm the minimum intensity required for the response. Throughout the experiment, the participants were asked to describe the sensation felt, express the degree of unpleasantness, and if pain, then quantify it on an 11-point scale VAS.

Chronic low back pain patients all naive to the procedure, were informed about the test procedure in details. The test was conducted in a silent environment with the subjects in overnight fasting state. The instructions and the technique were described before proceeding for the recordings. Subjects were then also equipped with a leaflet indicating Visual analogue scale ratings (VAS), from 0 to 10, 0-marked “no pain” and 10-“worst pain imaginable” at either end. The amplitude of the stimulation was manually controlled and recordings were performed with a Biopac EMG System. (Model: BSLSTMA, Biopac systems, Inc. Santa Barbara, California).

The characteristic EMG response with latency between 80 and 180 msec, duration between 40 and 60 msec, amplitude >30–40 mV was considered. Three records for each of the parameters; NFR threshold, latency, amplitude, duration, and area under the curve, were averaged for each patient.

**Figure 1:**
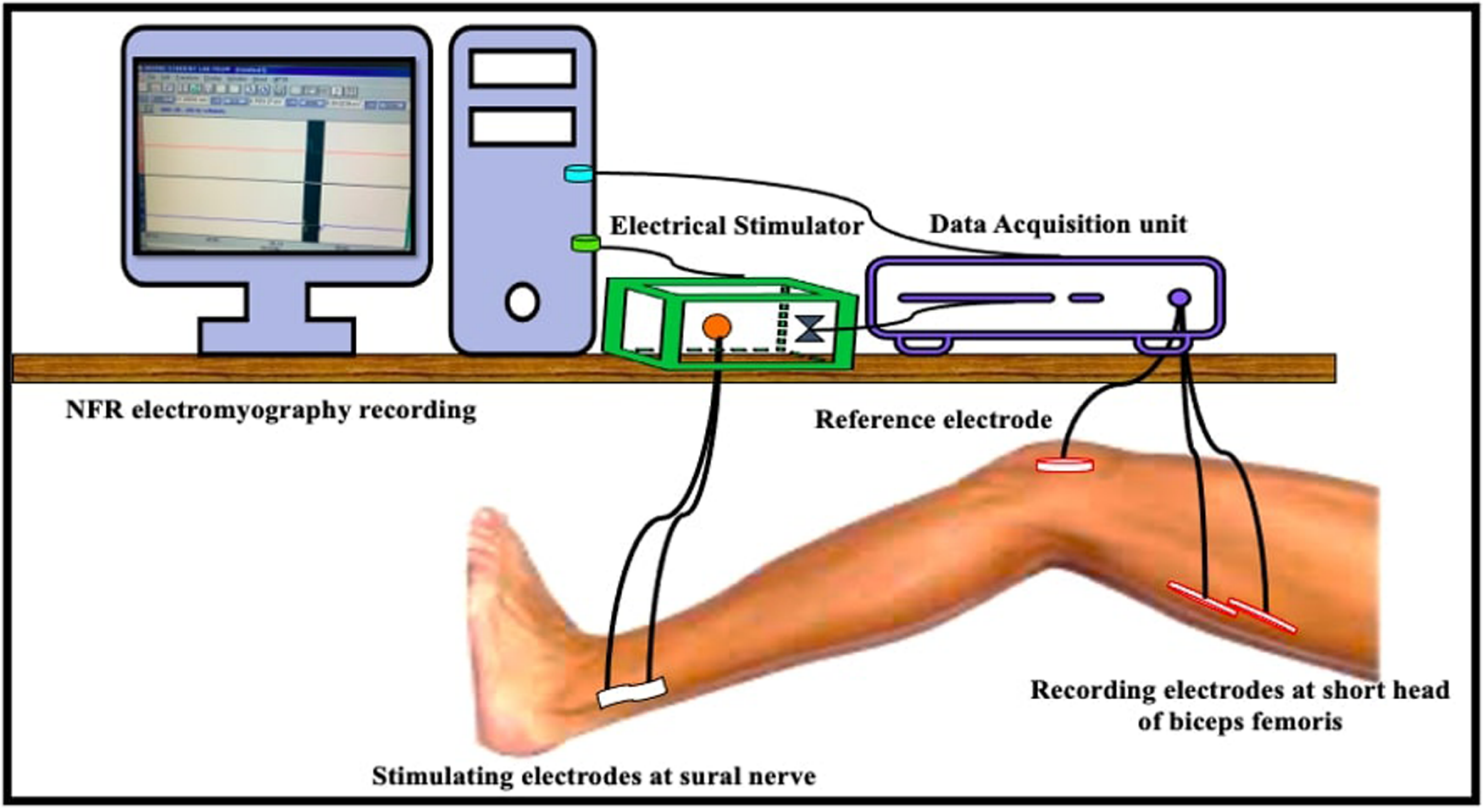
Diagrammatic representation of set-up for nociceptive flexion reflex recording (NFR electromyography recording, elecrical stimulator, data acquisition unit, stimulation, reference and recording electrodes at their respective sites).

For Diffuse Noxious Inhibitory Control testing (Conditioned pain modulation) a test and conditioning stimuli were used. The test stimulus was nociceptive flexion reflex (described earlier) and the conditioning stimulus was noxious cold water immersion of the contralateral hand. Each experimental session had six time-epochs: during immersion (max 90 s), and after 1 min of hand removal until 5 min (T1–T5) explain properly. Three stimuli were delivered within each time epoch and were averaged to give a representative nociceptive flexion reflex threshold Value (Figure 1).

### Medical yoga therapy yoga protocol

The orientation and execution of yoga protocol was conducted by a qualified yoga therapist five days a week for one month at Integral Health Clinic, Department of Physiology, All India Institute of Medical Sciences (AIIMS), New Delhi, India. The protocol was a comprehensive, yoga-based intervention program lasting for 2 h per day for 5 days a week for a total of 4 weeks. It consists of an integrated and pretested intervention (35, 36) comprising of theory and practice sessions. To ensure the quality of the program and to ensure that the participants get enough time with the expert, only 6–8 participants were assigned to the program at a given point of time.

**Figure 2:**
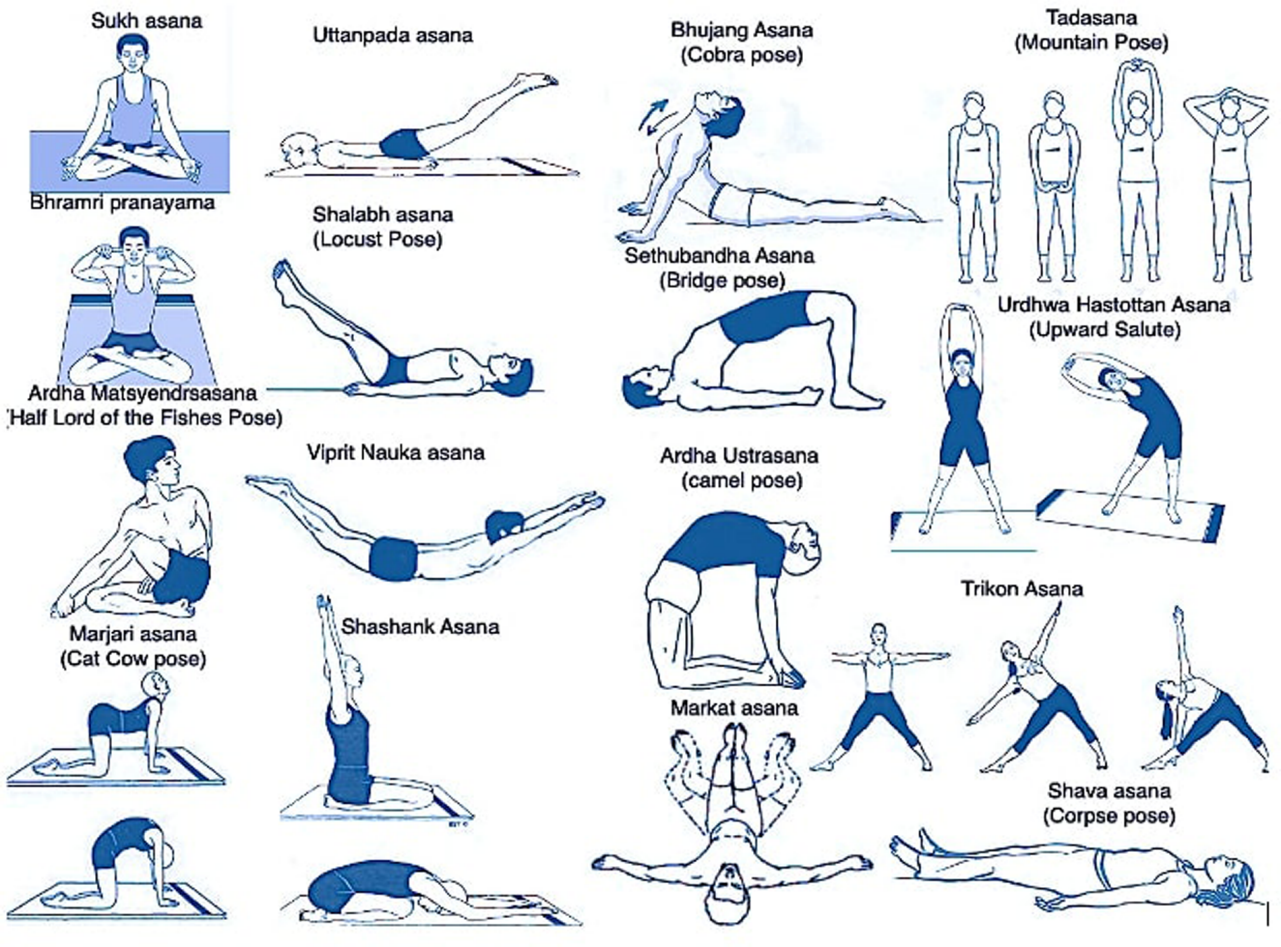
Medical Yoga therapy (Asanas).

The Patients were advised to follow same yoga protocol at home for same duration for another 4 weeks. They were advised to maintain a diary in which they would mention about yoga practice done or not, number and frequency of medication taken and were in constant touch with the experts (Table 1; Figure 2).

**Table 1:** Medical yoga therapy protocol.

Asana	Frequency
Loosening exercises/SukshmaVyayama	
Griva ShaktiVikasaka/Neck Movement (Neck Rotation clockwise, anti-clockwise, up and down, sideways)	6 times each
BhujaValli ShaktiVikasaka Purna Bhuja ShaktiVikasaka/Shoulder Movement (Shoulder rotation clockwise, anti-clockwise, Elbows fold in and out, Rotate arms alternatively)	6 times each
Kati ShaktiVikasaka (I, II, III, IV,V)/Trunk Movement (Twist body at waist, Sideways bend-alternate sides)	6 times each
Jangha ShaktiVikasaka (Il-A&B)/Thigh Movement (chair pose, kick with alternate leg)	6 times each
Janu Shakti Yikasaka/Knee movement (Rotate knees clockwise, anti-clockwise)	6 times each
Pada-mula shaktiVikasaka/Ankle movement (Toes pointing outwards-up, down, sideways)	6 times each
Gulpha-pada-pristha-pada-tala shaktiVikasaka/Ankle movement (Rotate ankle clockwise and anti-clockwise)	6 times each
Standing Asanas	3 times each with regulated breathing
Tadasana	
Triyak Tadasana	
Trikonasana	
Samkonasana	
Sitting Asanas	3 times each with regulated breathing
Shashakasana	
Paschimaanasana	
Seubandhasana	
Marjariasana	
Lying on stomach Asanas	3 times each with regulated breathing
Makarasana	
Bujangasana	
Shalabhasana	
Lying on back Asanas	3 times each with regulated breathing
Pavanmuktasana	
Sarvangasana	
Setubandhasana	
Markatasana	
Deep relaxation/Shavasana	5 min
Pranayam	15 min
Bhramari	
AnulomVilom	
Turn to side and sit up, End with OM chanting in a mediative posture	For 5 min

## Results

Chronic low back pain patients were randomly allocated to Medical Yoga Therapy (MYT) and Standard Care Therapy (SCT) group (*n* = 58 and *n* = 50 respectively). At baseline both the groups were comparable, we recorded all the parameters of the study at baseline for 98 CLBP patients (50 in MYT group and 48 in SCT group), at end of 4 weeks (48 in MYT group and 45 in SCT group), and at end of 8 weeks (45 in MYT group and 40 in SCT group). Mean age of patients was 42.5 ± 12.6 years. General demographic and physiological parameters comprising of age, height, body weight, body mass index, blood pressure, heart rate, duration of pain, medication intake/week were recorded at all time points. Quality of life was assessed using WHOQOLBREF questionnaire, and McGill pain questionnaire and VAS rating were used for subjective reporting of pain. Nociceptive flexion reflex and diffuse noxious inhibitory control test were recorded to objectively assess the effect of both the interventions in CLBP patients. Following tables show the data analysed and comparisons drawn between MYT and SCT groups and within groups at baseline and at end of 4 and 8 weeks of both interventions.

Physiological parameters of patients' i.e., body weight, body mass index, heart rate and blood pressure reduced significantly after 4 weeks of Medical Yoga therapy. Body weight reduced significantly at the end of both 4 and 8 weeks of yogic intervention. Whereas CLBP patients in the Standard Care therapy group did not show any significant change in body weight. Body mass index of CLBP patients in Medical Yoga therapy group were also significantly lower than Standard Care therapy group at both time points.

We noted the medication intake frequency per week for both the groups and found CLBP patients in Medical Yoga therapy group took significantly lesser amount of pain-relieving medications/week (1.65 ± 0.85), compared to Standard Care therapy group (5.98 ± 2.01), *p *= < 0.0001*, at end of 4 weeks of interventions.

**Figure 3:**
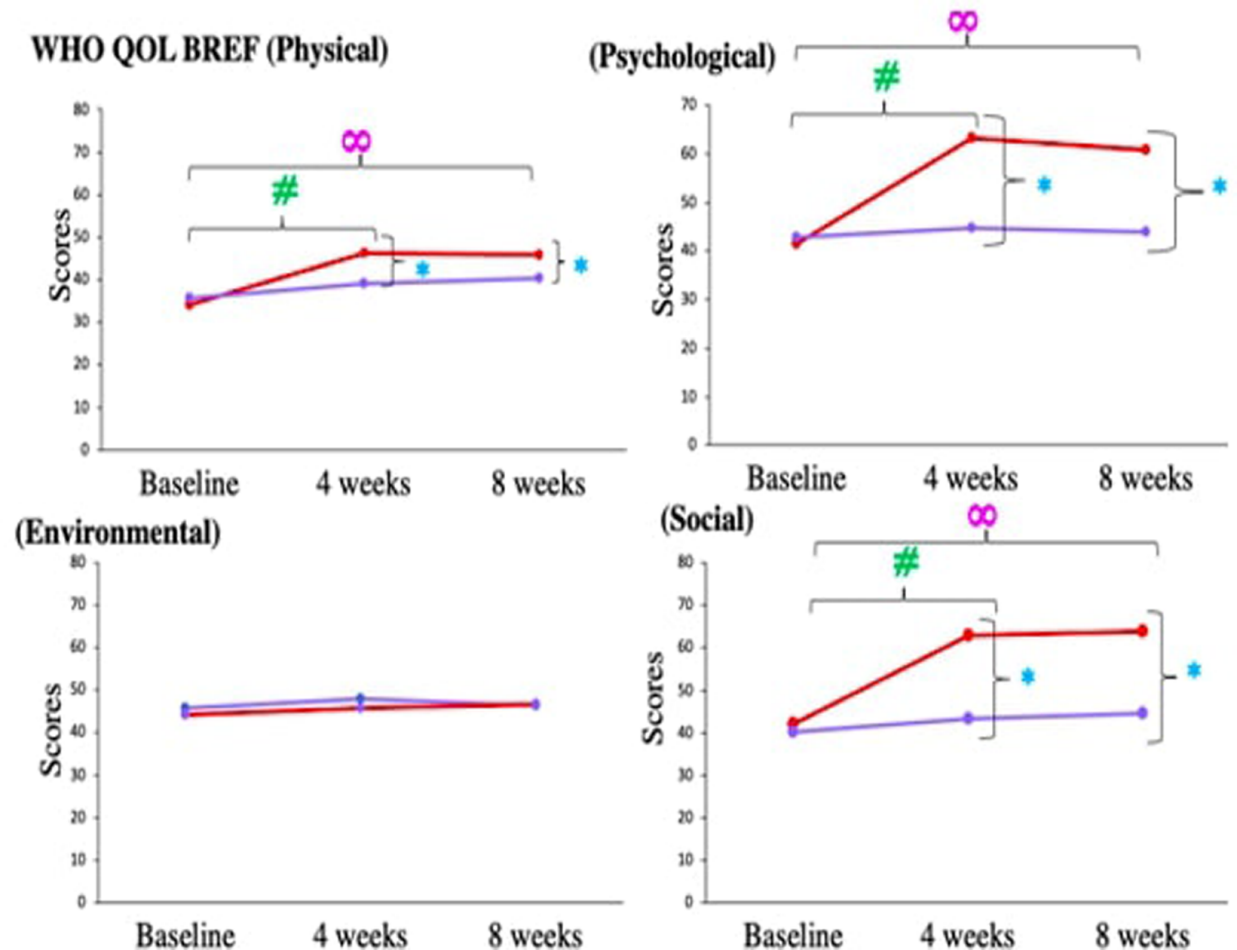
Comparison of WHOQOL BREF components in chronic low back pain patients between medical yoga therapy (red) and standard care therapy (purple) at baseline, at 4 weeks and at 8 weeks. Level of significance is set at 5% and denoted by *(comparison between Medical Yoga therapy and Standard Care therapy), #(baseline vs. 4 weeks) ∞(baseline vs. 8 week).

We found heart rate and systolic and diastolic blood pressure were reduced significantly after 4 weeks of Medical Yoga therapy, whereas after Standard Care therapy they remain unaltered. (Table 2). At the end of 8 weeks, Heart rate and blood pressure of CLBP patients in the Medical Yoga therapy group were significantly reduced to 75.39 ± 4.37 beats/min (*p *= < 0.0001∞), and 123.7 ± 6.1/77.9 ± 4.9 mm Hg; *p *= < 0.0001∞). In the Standard Care therapy group, we did not find any significant change in either heart rate or blood pressure (Table 2). We found significant improvement in overall quality of life assessed by WHOQOL BREF questionnaire, after 4 and 8 weeks of yogic intervention (*p *= < 0.0001#) (Table 3 and Figure 3).

**Table 2:** Represents comparison of general physiological parameters between medical yoga therapy and standard care therapy groups at 3 time points (baseline, at 4 weeks and at 8 weeks of intervention in chronic low back pain patients.

	Baseline	4 weeks	8 weeks	*p*-value	
Parameter	Mean ± SD	Mean ± SD	Mean ± SD	Baseline vs. 4 wks	Baseline vs. 8 wks
Weight (Kg)					
MYT	68.86 ± 8.3	64.06 ± 7.3	65.13 ± 8.55	0.001^#^	0.001^∞^
SCT	69.76 ± 5.5	71.6 ± 3.8	70.87 ± 5.96	0.091	0.107
PValue (bet)	0.082	<0.0001*	<0.0001*		
Height (cm)					
MYT	158.5 ± 5.5				
SCT	159.5 ± 7.3				
*p*-value (bet)	0.974				
Body mass index (BMI)					
MYT	30.44 ± 4.97	28.43 ± 3.86	28.12 ± 3.09	<0.0001	<0.001
SCT	30.65 ± 2.9	31.21 ± 4.84	31.04 ± 5.03	0.098	0.10
*p*-value (bet)	0.113	<0.0001*	< 0.0001*		
Medication intake/week					
MYT	5.5 ± 1.98	1.65 ± 0.85	2.08 ± 1.01	0.001^#^	0.001^∞^
SCT	6.08 ± 1.87	5.98 ± 2.01	4.97 ± 2.98	0.096	0.071
*p*-value (bet)	0.092	<0.0001*	<0.0001*		
Systolic BP (mm Hg)					
MYT	128.5 ± 5.8	123.4 ± 4.98	123.7 ± 6.1	<0.0001	<0.0001
SCT	126.6 ± 4.6	127.7 ± 6.6	126.6 ± 5.8	0.098	0.143
*p*-value (bet)	0.0517	0.0002*	0.0039*		
Diastolic BP (mm Hg)					
MYT	82.1 ± 4.1	76.5 ± 3.54	77.9 ± 4.9	<0.0001	<0.0001
SCT	84.6 ± 5.3	83.43 ± 6.7	82.43 ± 2.7	0.085	0.078
*p*-value (bet)	0.713	< 0.0001*	< 0.0001*		
Heart Rate (beats/min)					
MYT	89.5 ± 10.5	73.65 ± 3.89	75.39 ± 4.37	<0.0001^#^	<0.0001^∞^
SCT	88.73 ± 9.1	86.48 ± 5.83	87.5 ± 5.3		
*p*-value (bet)	0.3271	< 0.0001*	< 0.0001*		

* indicates comparison between parameters between Medical Yoga Therapy and Standard Care Therapy.

∞ indicates comparison between baseline and 8 weeks of Medical Yoga Therapy.

# indicates comparison between baseline and 4 weeks of Medical Yoga Therapy.

**Table 3:** Shows comparison of quality of life assessed by WHOQOL BREF questionnaire in chronic low back pain patients between medical yoga therapy and standard care therapy at baseline, at 4 weeks and at 8 weeks.

	Baseline	4 weeks	8 weeks	*p*-value	
Parameter	Mean ± SD	Mean ± SD	Mean ± SD	Baseline vs. 4 wks	Baseline vs. 8 wks
WHOQOL (BREF) Physical					
MYT	34.1 ± 17.6	46.4 ± 15.7	45.9 ± 10.8	<0.0001^b^	<0.0001^c^
SCT	35.8 ± 12.5	39.2 ± 11.8	40.2 ± 10.8	0.04^b^	0.03^c^
*p*-value (bet)	0.2176	<0.001^a^	<0.0001^a^		
WHOQOL (BREF) Psychological					
MYT	41.4 ± 19.2	63.2 ± 16.3	60.87 ± 12.3	<0.0001^b^	<0.0001^c^
SCT	42.9 ± 11.3	44.7 ± 13.2	43.9 ± 10.2	0.1	0.098
*p*-value (bet)	0.312	<0.001^a^	<0.001^a^		
WHOQOL (BREF) Environmental					
MYT	45.6 ± 18.6	47.8 ± 16.6	46.49 ± 14.5	0.156	0.16
SCT	44.3 ± 9.98	45.7 ± 10.5	46.7 ± 7.75	0.064	0.084
*p*-value (bet)	0.075	0.1513	0.087		
WHOQOL (BREF) Social					
MYT	42.0 ± 13.2	62.58 ± 15.8	63.87 ± 10.2	<0.0001^b^	<0.0001^c^
SCT	40.3 ± 15.4	43.2 ± 11.87	44.6 ± 10.7	0.053	0.046^c^
*p*-value (bet)	0.068	<0.001^a^	<0.001^a^		

Level of significance is set at 5% and denoted by:

^a^ (comparison between Medical Yoga therapy and Standard Care therapy).

^b^ (baseline vs. 4 weeks)

^c^ (baseline vs. 8 week).

**Figure 4:**
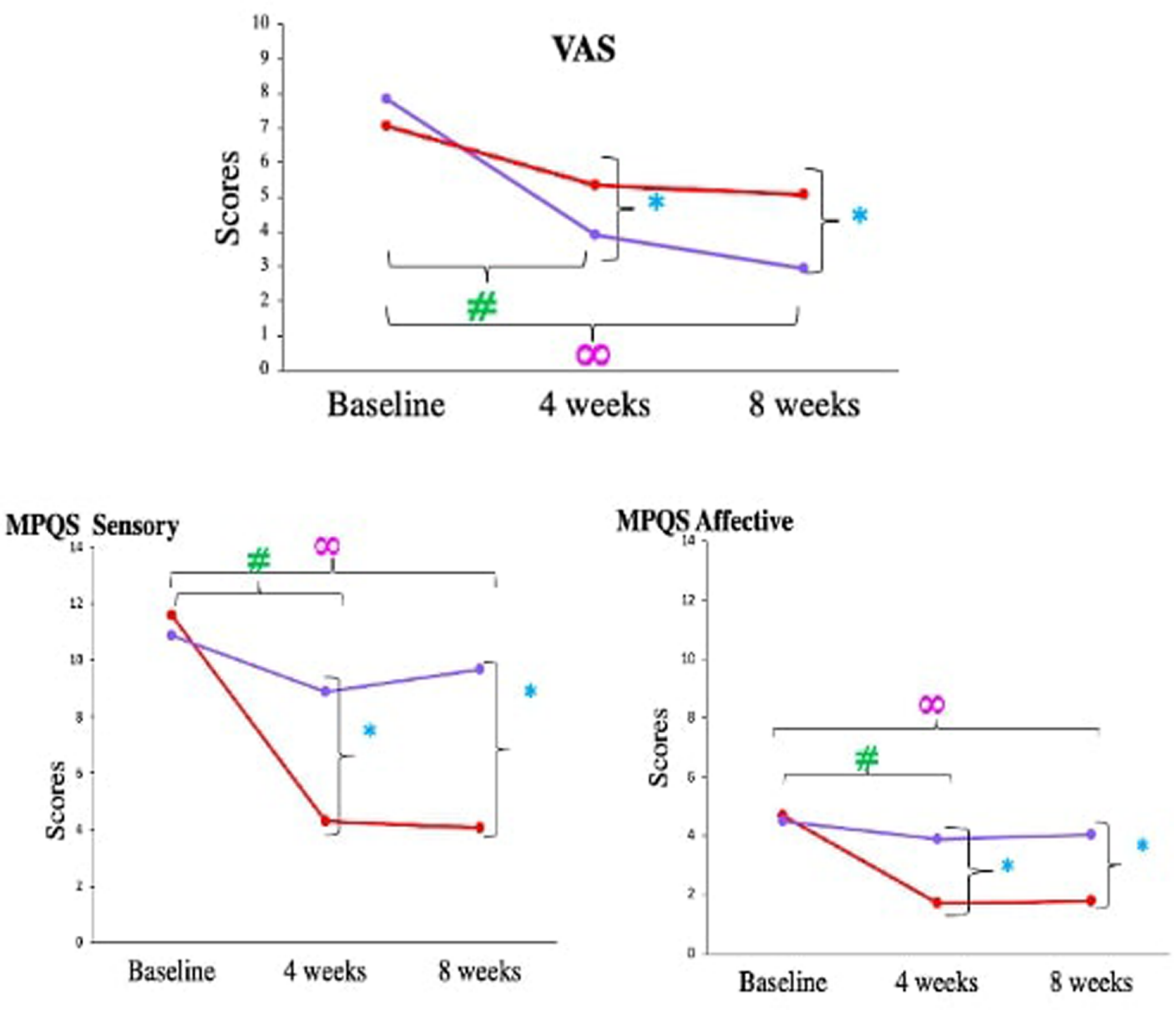
Comparison of VAS scores and short form McGrill pain questionnaire components in chronic low back pain patients between medical yoga therapy and standard care therapy at baseline, at 4 weeks and at 8 weeks. Level of significance is set at 5% and denoted by *(comparison between Medical Yoga therapy and Standard Care therapy), #(baseline vs. 4 weeks) ∞(baseline vs. 8 week).

Visual analogue scale (VAS) scores for patients in both the groups were comparable at baseline, but subjective pain rating decreased significantly more after 4 of Medical Yoga therapy compared to Standard Care therapy (Medical Yoga therapy group-3.92 ± 2.04, Standard Care therapy- 5.35 ± 3.32, *p *= < 0.0001*). Both sensory and affective component scores of McGill Pain questionnaire revealed significantly more reduction in pain experience in Medical Yoga therapy group compared to Standard Care therapy. (Sensory; Medical Yoga therapy- 4.3 ± 3.7, Standard Care therapy- 8.9 ± 3.2, *p* = 0.0043*, Affective; Medical Yoga therapy- 1.7 ± 1.6, Standard Care therapy-3.9 ± 1.32, *p* = 0.002*. At the end of 8 weeks, the subjective pain scores of CLBP patients in Medical Yoga therapy group were 2.95 ± 1.01, which was significantly lower than Standard Care therapy 5.06 ± 2.07 (*p* = 0.005*). Both sensory and affective component scores of McGill Pain questionnaire revealed significantly more reduction in pain experience in Medical Yoga therapy group compared to Standard Care therapy. (Sensory; Medical Yoga therapy- 4.07 ± 4.5, Standard Care therapy- 9.7 ± 3.6, *p* = 0.005*, Affective; Medical Yoga therapy- 1.8 ± 1.38, Standard Care therapy-4.03 ± 2.2, *p *= < 0.0001* (Table 4 and Figure 4).

**Table 4:** Shows comparison of pain status in chronic low back pain patients between medical yoga therapy and standard care therapy at baseline, at 4 weeks and at 8 weeks.

	Baseline	4 weeks	8 weeks	*p*-value	
Parameter	Mean ± SD	Mean ± SD	Mean ± SD	Baseline vs. 4 wks	Baseline vs. 8 wks
Subjective (VAS)					
MYT	7.82 ± 3.06	3.92 ± 2.04	2.95 ± 1.01	<0.0001^b^	<0.0001^c^
SCT	7.08 ± 2.7	5.35 ± 3.32	5.06 ± 2.07	0.005^b^	0.0023^c^
*p*-value (bet)	0.15	<0.0001^a^	0.005^a^		
MPQ-Sensory					
MYT	11.6 ± 8.5	4.3 ± 3.7	4.07 ± 4.5	<0.0001^b^	<0.0001^c^
SCT	10.9 ± 9.3	8.9 ± 3.2	9.7 ± 3.6	0.094	0.13
*p*-value (bet)	0.513	0.0043^a^	0.0075^a^		
MPQ-Affective					
MYT	4.7 ± 3.6	1.7 ± 1.6	1.8 ± 1.38	<0.0001^b^	<0.0001^c^
SCT	4.5 ± 4.1	3.9 ± 1.32	4.03 ± 2.2	0.086	0.094
*p*-value (bet)	0.12	0.002^a^	<0.0001^a^		

Level of significance is set at 5% and denoted by:

^a^ (comparison between Medical Yoga therapy and Standard Care therapy).

^b^ (baseline vs. 4 weeks).

^c^ (baseline vs. 8 week).

**Figure 5:**
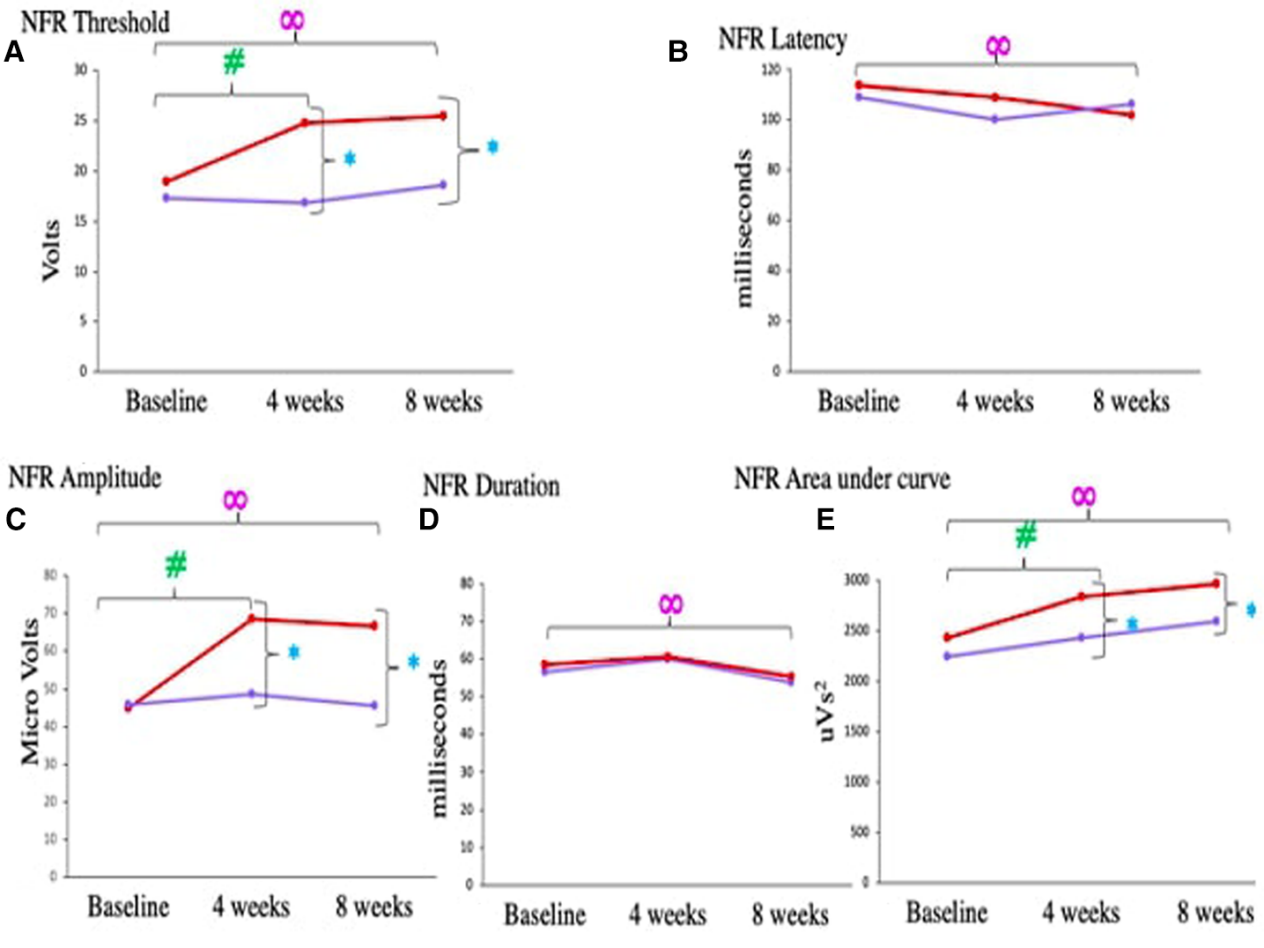
Comparison of (**A**) NFR threshold, (**B**) NFR latency, (**C**) NFR amplitude, (**D**) NFR duration, (**E**) NFR area under curve, in chronic low back pain patients between medical yoga therapy and standard care therapy at baseline, at 4 weeks and at 8 weeks. Level of significance is set at 5% and denoted by *(comparison between Medical Yoga therapy and Standard Care therapy), #(baseline vs. 4 weeks) ∞(baseline vs. 8 week).

We assessed pain objectively by recording the NFR response in both the groups at all time points. The baseline NFR thresholds for both the groups were comparable 18.91 ± 4.55 V (Medical Yoga therapy group) and 17.31 ± 4.64 V (Standard Care therapy group), *p* = 0.089. Nociceptive Flexion Reflex threshold increased significantly in Medical Yoga therapy group (24.8 ± 4.52 V, *p *= < 0.0001#), which differed significantly from 4 weeks of Standard Care therapy (16.18 ± 3.3 V, *p *= < 0.0001*). NFR latencies were comparable between the groups across all time points. NFR amplitude significantly increased from 44.87 ± 6.23 mV to 68.66 ± 7.7 mV after 4 weeks of yogic intervention (*p *= < 0.0001#), but after 4 weeks of Standard Care therapy the amplitudes were comparable, also there was significant difference when compared to Medical Yoga therapy group (*p *= < 0.0001*). NFR duration did not change over time and across the groups. Area under the curve for NFR response increased significantly from 2244.46 ± 436.2 mVs (baseline), to 2430.6 ± 80 mVs (after 4 weeks of Medical Yoga therapy therapy), (*p* = 0.004#) (Table 5 and Figure 5). Nociceptive Flexion Reflex threshold increased significantly in Medical Yoga therapy group (25.44 ± 3.4 V, *p *= < 0.0001∞) which differed significantly from 8 weeks of Standard Care therapy (18.54 ± 5.4 V, *p *= < 0.0001*). As far as NFR latency is considered there was a significant decrease after 8 weeks of yogic intervention (baseline- 113.75 ± 13.5 ms, at 8 weeks- 101.9 ± 6.89 ms, *p* = 0.0001∞). After 8 weeks of interventions in NFR amplitude in Medical Yoga therapy group was 66.76 ± 10.5 mV, significantly higher than 45.6 ± 8.97 mV, *p *= < 0.0001* in Standard Care therapy group. Area under the curve for NFR response increased significantly from 2244.46 ± 436.2 mVs (baseline) and to 2598.86 ± 68 mVs (after 8 weeks of Medical Yoga therapy, (*p* = 0.001∞), whereas after 4 and 8 weeks of Standard Care therapy, there were no significant differences in the areas.

**Table 5:** Shows comparison of nociceptive flexion reflex parameters NFR threshold, latency, amplitude, duration and area under curve between medical yoga therapy and standard care therapy groups at 3 time points (baseline, at 4 weeks and at 8 weeks of intervention in chronic low back pain patients. Data is checked for normality using Shapiro-Wilk test, data is parametric represented as Mean ± Standard deviation. Comparison between groups is done using unpaired t test and within the group temporally is done using one way ANOVA.

	Baseline	4 weeks	8 weeks	*p*-value	
Parameter	Mean ± SD	Mean ± SD	Mean ± SD	Baseline vs. 4 wks	Baseline vs. 8 wks
NFR Threshold (V)					
MYT	18.91 ± 4.5	24.8 ± 4.52	25.44 ± 3.4	<0.0001^b^	<0.0001^c^
SCT	17.31 ± 4.64	16.18 ± 3.3	18.54 ± 5.4	0.096	0.083
*p*-value (bet)	0.089	<0.0001^a^	<0.0001^a^		
NFR Latency (ms)					
MYT	113.7 ± 13.5	108.9 ± 11	101.9 ± 6.8	0.0807	0.0001^c^
SCT	109.2 ± 15.5	104.3 ± 17	106 ± 15.9	0.0058	0.074
*p*-value (bet)	0.1611	0.07	0.0807		
NFR Amplitude (mV)					
MYT	44.87 ± 6.3	68.66 ± 7.7	66.7 ± 10.5	<0.0001^b^	<0.0001^c^
SCT	45.7 ± 6.1	48.6 ± 9.76	45.6 ± 8.97	0.064	0.18
*p*-value (bet)	0.98	<0.0001^a^	<0.0001^a^		
NFR Duration (ms)					
MYT	56.55 ± 6.7	58.85 ± 7.9	53.8 ± 6.75	0.07	0.03^c^
SCT	58.55 ± 8.7	60.58 ± 8	55.4 ± 10.7	0.063	0.04^c^
*p*-value (bet)	0.074	0.14	0.084		
NFR AUC (mVs)					
MYT	2244.46 ± 43	2430.6 ± 8	2598.86 ± 6	0.004^b^	0.001^c^
SCT	2435.5 ± 45	2744.8 ± 6	2974 ± 59.4	0.01^b^	0.009
*p*-value (bet)	0.075	<0.0001^a^	<0.0001^a^		

Level of significance is set at 5% and denoted by:

^a^ (comparison between Medical Yoga therapy and Standard Care therapy).

^b^ (baseline vs. 4 weeks).

^c^ (baseline vs. 8 week).

**Figure 6:**
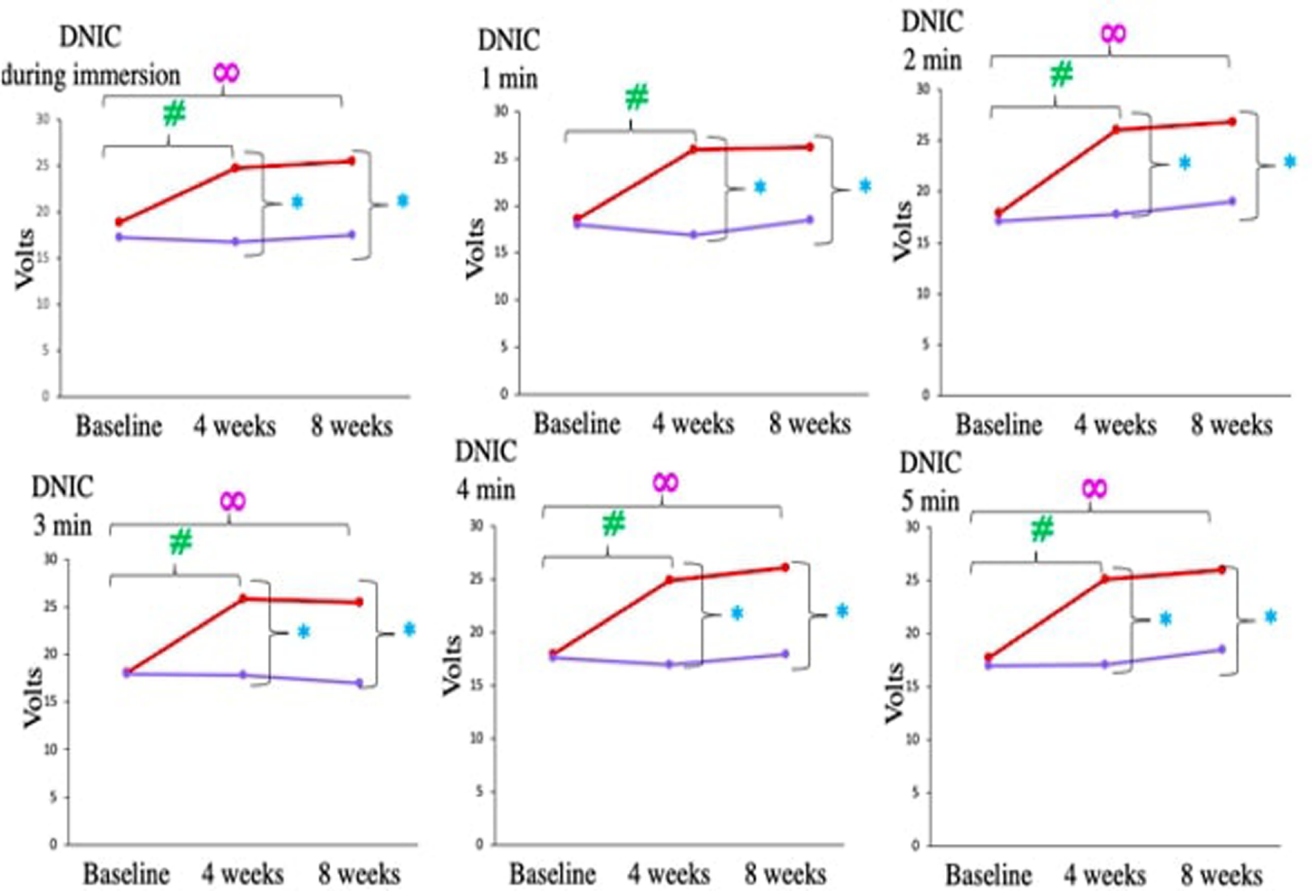
Comparison of diffuse noxious inhibitory control (cold pressor test) during immersion and at end 1–5 min, in chronic low back pain patients between medical yoga therapy and standard care therapy at baseline, at 4 weeks and at 8 weeks. Level of significance is set at 5% and denoted by *(comparison between Medical Yoga therapy and Standard Care therapy), #(baseline vs. 4 weeks) ∞(baseline vs. 8 week).

Diffuse noxious inhibitory controls/conditioned pain modulation assessed by cold pressor test revealed that Medical Yoga therapy intervention improved the descending pain modulation in CLBP patients. This is supported by the following findings, DNIC during immersion, at baseline NFR threshold was 18.91 ± 4.55 V, which increased significantly to 24.8 ± 4.52 V, *p *= < 0.001# after 4 weeks. There was a significant increase in NFR thresholds at all time points (from 1 min to 5 min) in the Medical Yoga therapy group at the end of 4 weeks. But after Standard Care therapy no such increase was observed, at any of the 5 time points of Cold pressor test (1 min to 5 min) or across 3 observation points (Table 6 and Figure 6) NFR threshold during immersion increased to 25.44 ± 3.41 V after 8 weeks *p *= < 0.001∞. There was a significant increase in NFR thresholds at all time points (from 1 min to 5 min), in the Medical Yoga therapy group at the end of 8 weeks. (Table 5 and Figure 5). In the Standard Care therapy group we did not find an increase, at any of the 5 time points of the cold pressor test (1 min to 5 min) or across 3 observation points (Table 6 and Figure 6).

**Table 6:** Shows comparison of DNIC assessed by CPT between medical yoga therapy and standard care therapy groups at 3 time points (baseline, at 4 weeks and at 8 weeks) in chronic low back pain patients. Data is checked for normality using Shapiro-Wilk test, data is parametric represented as Mean ± SD. Comparison between groups is done using unpaired t test and within group temporally is done using one way ANOVA.

	Baseline	4 weeks	8 weeks	*p*-value	
Parameter	Mean ± SD	Mean ± SD	Mean ± SD	Baseline vs. 4 wks	Baseline vs. 8 wks
DNIC (V)					
MYT	18.91 ± 4.55	24.8 ± 4.52	25.44 ± 3.41	<0.001^b^	<0.001^c^
SCT	17.31 ± 4.6	16.18 ± 3.36	17.54 ± 4.2	0.096	0.103
*p*-value (bet)	0.1311	<0.0001^a^	<0.0001^a^		
I min (V)					
MYT	18.55 ± 5.32	25.98 ± 3.32	26.21 ± 4.12	<0.001^b^	<0.01^c^
SCT	17.98 ± 5.5	16.84 ± 5.32	18.52 ± 4.1	0.074	0.079
*p*-value (bet)	0.04	<0.0001^a^	<0.0001^a^		
2 min (V)					
MYT	17.96 ± 4.76	26.01 ± 4.27	26.89 ± 3.97	<0.001^b^	<0.01^c^
SCT	17.09 ± 4.4	17.76 ± 4.21	18.98 ± 4.08	0.109	0.083
*p*-value (bet)	0.104	<0.0001^a^	<0.0001^a^		
3 min (V)					
MYT	18.11 ± 2.9	25.87 ± 3.2	25.48 ± 2.98	<0.001^b^	<0.001^c^
SCT	17.95 ± 3.4	17.87 ± 3.25	16.95 ± 3.9	0.195	0.08
*p*-value (bet)	0.16	<0.0001^a^	<0.0001^a^		
4 min (V)					
MYT	17.90 ± 3.	24.87 ± 4.1	26.05 ± 3.68	<0.001^b^	<0.001^c^
SCT	17.6 ± 4.87	16.98 ± 3.87	17.97 ± 4.9	0.096	0.16
*p*-value (bet)	0.14	<0.0001^a^	<0.0001^a^		
5 min (V)					
MYT	17.69 ± 3.8	25.09 ± 3.25	25.95 ± 2.89	<0.001^b^	<0.01^c^
SCT	16.98 ± 5.2	17.02 ± 2.89	18.43 ± 3.1	0.088	0.005^c^
*p*-value (bet)	0.108	<0.0001^a^	<0.0001^a^		

Level of significance is set at 5% and denoted by:

^a^ (comparison between Medical Yoga therapy and Standard Care therapy).

^b^ (baseline vs. 4 weeks).

^c^ (baseline vs. 8 weeks).

## Discussion

The aim was to objectively assess the effect of Medical Yoga Therapy on chronic low back pain, using a spinally mediated reflex response, NFR and descending pain modulation paradigm. Also, we compared the effect of MYT with Standard care therapy on pain (subjectively-VAS, quality of life, sensory and affective components, and objectively by recording NFR and DNIC response) at the end of 4 and 8 weeks of both interventions. There was a significant reduction in body weight, body mass index, heart rate and blood pressure in MYT group, compared to SCT group both at end of 4 and 8 weeks. We found significant reduction in heart rate after yogic intervention which is supported by a study of 8-week of moderate intensity aerobic exercise intervention, and the authors reported similar results (37). However, the basic mechanism of the two interventions is different. The postulated mechanism of action of Medical Yoga therapy is through parasympathetic activation of autonomic nervous system and the associated stress relieving mechanisms in the body which is substantiated by our finding of significantly decreased blood pressure and heart rate after Medical Yoga therapy. Medical Yoga therapy blunts the physiological response to stress by enhancing GABA-mediated cortical inhibitory tone (38). Yoga increases the blood flow and nutrients to the soft tissues in the back region, improve the healing process and reduce stiffness that results in back pain (39). Therapeutically Medical Yoga therapy helps people manage health condition and reduce the pain symptoms (International Association of Medical Yoga therapy Therapists, 2016). Also, an increase in parasympathetic tone is suggested by a significant decrease in systolic and diastolic blood pressure and heart rate after yogic intervention in our study. Pain can be assessed subjectively by Visual analogue scale and short form of McGill pain questionnaire. Chronic low back pain patients had moderate to severe pain at baseline VAS (7.82 ± 3.06) which significantly reduced to (1.92 ± 2.04) after Medical Yoga therapy. Williams et al., in 2009 reported significant reduction in pain scores after 12 weeks of yogic intervention. It is delineated that MYT affects emotional aspects of chronic pain, reduces anxiety and depression effectively and improves the quality of life (40). The literature suggests a significant number of studies that have reported significant reduction in VAS scores, body weight, blood pressure and other subjective measures of assessment (13, 21, 41, 15, 12, 19, 20, 18, 42, 14). Medical Yoga therapy helps relax, energize, remodel and strengthen the body and psyche and starts a “relaxation response” of the neuroendocrinal axis (43). Yoga's benefits were largely attributable to the physical benefits of stretching and strengthening of the muscles. It is also suggested that parasympathetic activation is necessary for hypoalgesia, which is also reported in our study (significant reduction in blood pressure and heart rate). Hölzel BK et al., in 2010 reported subjects who meditated 30 min a day for eight weeks had a reduction of grey matter in the amygdala—which is linked to fear, anxiety and emotion. MYT increases activity of limbic system that alleviates mood and decrease anxiety (44).

Nociceptive Flexion Reflex is a widely used and accepted tool to assess the objective pain perception and its modulation at spinal level. NFR recordings at baseline revealed CLBP patients had significant hyperalgesia as the threshold was significantly lower (18.91 ± 4.55 V) compared to age and gender matched healthy controls (28.95 ± 3.75 V, *p *= < 0.0001). An 8 week MYT, resulted in increase in the NFR thresholds significantly, indicating significant reduction in pain. Asanas are isometric exercises that enhance steadiness of the body and optimize body functioning. The ability to perform asanas even in chronic pain boosts the self-confidence, as it positively reinforces the patient to perform farther and better. The coupled relaxation breaks the chronic pain cycle and reverses the pain reinforcing forces. MYT increases local blood circulation that washes out the inflammatory mediators which relieves sensitization of the nociceptors. Van der Hulst M et al., 2010 reported increased paraspinal electromyographic (EMG) activity in CLBP (45). Wrong postures and sedentary lifestyle causes wasting and weakness of postural muscles, leading to functional disability and chronicity of pain. Asanas like Pavanamuktasana, Bhujangasana, Shalabhasana etc help in controlled and coupled activation and relaxation of the spinal muscles such as superficial and deep back muscles (Erector spinae, Intertransversarii, Interspinalis, Multifidi, Semispinalis, Splenius capitals and Longisimus, Serratus posterior superior). Thus Medical Yoga therapy increases sensory inputs from peripheral proprioceptors to the cortical areas which also modulates motor activity. Chronic pain can lead to either inefficient descending pain modulation or aggravated ascending pain facilitation. Diffuse noxious inhibitory controls test is a tool to assess the integrity of descending pain modulatory pathways. To further explore the role of descending pain modulation in CLBP patients, DNIC test was performed and it revealed CLBP patients poor descending inhibitory controls as their NFR thresholds remained unchanged during and after cold pressor test. But after MYT their DNIC improved as suggested by significant increase in their NFR thresholds during and after CPT. This can be attributed to the fact that MYT helps in relieving stress and also decrease inflammation thus decreasing central sensitization responsible for pain aggravation. To the best of our knowledge there is no study that has objectively assessed effect of MYT on such aspects of pain, and so no reports exist to support our findings for NFR response after yogic intervention in CLBP patients. Also to the best of our knowledge no study has utilized DNIC test as an objective assessment tool after yogic intervention in CLBP patients. NFR and CPT both tests can be widely used not only to assess pain objectively in chronic pain patients but can also act as a prognostic tool and determine the course of the disease. These tests can also be utilized in clinical setups to determine the effect of any therapeutic intervention without any subjective bias.

## Conclusion

It is well known that MYT is beneficial in alleviating pain and improving quality of life and parasympathetic activity in CLBP patients. But through this study we objectively assessed effect of MYT by Nociceptive flexion reflex and diffuse noxious inhibitory control tests in CLBP patients. We conclude that Medical Yoga Therapy surely does improve pain and descending pain modulation, reduces hyperalgesia in CLBP patients. We suggest NFR and DNIC tests should be routinely used as an objective assessment tool not only for MYT but also any other therapeutic interventions.

## Data Availability

The original contributions presented in the study are included in the article/Supplementary Material, further inquiries can be directed to the corresponding author/s.
